# Strategies to develop an LGBTQIA+-inclusive adolescent sexual health program evaluation

**DOI:** 10.3389/frph.2024.1327980

**Published:** 2024-03-22

**Authors:** Zabryna Balén, Emma Pliskin, Elizabeth Cook, Jennifer Manlove, Riley Steiner, Marisa Cervantes, Milagros Garrido, Claudia Nuñez-Eddy, Maeve Day

**Affiliations:** ^1^ Child Trends, Rockville, MD, United States; ^2^ Power to Decide, Washington, DC, United States; ^3^ MyHealthEd, Chapel Hill, NC, United States; ^4^ Healthy Teen Network, Churchville, MD, United States

**Keywords:** LGBTQ+  health, sex education, inclusive, sexual behavior, gender and sexuality, survey measures, recruitment, adolescent

## Abstract

**Introduction:**

Adolescent sexual health interventions are increasingly incorporating content that is inclusive of LGBTQIA+ youth (lesbian, gay, bisexual, transgender, queer/questioning, intersex, asexual, and other marginalized sexualities and genders). Evaluations of such programs must also be inclusive to enhance the validity of evaluation results and avoid further marginalization. We present strategies for increasing LGBTQIA+-inclusivity based on our evaluation of *SafeSpace,* a sexual health curriculum.

**Methods:**

To design an LGBTQIA+-inclusive program evaluation, we leveraged LGBTQIA+ research staff’s insights, pursued a parental consent waiver, developed an inclusive recruitment plan, and crafted demographic and sexual behavior survey measures with input from youth and equity experts. We conducted a pilot study with 42 youth ages 14–17 to assess the feasibility and efficacy of our strategies.

**Results:**

We obtained a parental consent waiver and recruited a majority LGBTQIA+ pilot study sample (62%). Using themes from cognitive interviews with youth and experts regarding inclusive framing and use of plain language, we refined demographic measures and expanded sexual behavior measures.

**Conclusion:**

Findings suggest that the strategies used to enhance LGBTQIA+-inclusivity in our evaluation of *SafeSpace* were effective in respectfully and more accurately capturing a fuller range of experiences and identities of LGBTQIA+ and cis-straight youth. The strategies and survey measures developed for this study can be applied to increase LGBTQIA+-inclusivity in other adolescent sexual health program evaluations.

## 1 Introduction

Comprehensive, evidence-based sexuality education^1^ has been shown to improve sexual and reproductive health (SRH) knowledge, behaviors, and outcomes for youth (1–4). However, there is growing recognition that sexuality education must be inclusive of youth who are lesbian, gay, bisexual, transgender, queer/questioning, intersex, asexual, and other marginalized sexualities and genders (LGBTQIA+^2^) (5), community-centered (6), and tailored to meet youth’s needs. An increasing percentage of U.S. youth identify as LGBTQIA+, representing one in four high school students in 2021 (7). And yet, the 2019 National School Climate Survey reports that only 8.2 percent of students had ever received LGBTQ +-inclusive sex education in school (8). Often, school-based sexual health curricula focus predominantly on the needs of heterosexual cisgender youth without discussion of sexual orientation and gender identity development. The curricula often exclude LGBTQIA+-inclusive examples of healthy relationships and consent. Additionally, programs typically emphasize penile-vaginal (PV) sex (9) and its associated pregnancy risk, while excluding protective practices related to other types of sexual activity particularly relevant to LGBTQIA+ youth.

There are significant consequences of inadequate sexuality education for LGBTQIA+ youth. Heteronormative and cisnormative sex education programs can alienate LGBTQIA+ youth and contribute to feelings of sexual shame and other negative mental health outcomes (10–13). Receiving sex education that is not inclusive is correlated with increased likelihood of experiencing sexual violence and engaging in sexual behaviors that increase risk for unintended pregnancy and sexually transmitted infections (STIs) (10, 12, 14). In fact, LGBTQIA+ young people assigned female at birth (AFAB) are more likely to experience unintended pregnancy than their heterosexual cisgender peers (15–18). Although less studied, evidence also suggests that trans masculine young people are at least as likely to become pregnant as cisgender young people (19). In addition to a lack of inclusive sex education, these disproportionate outcomes may be associated with experiences of stigma and discrimination (20, 21), lack of support (22), limited connectedness with family and school (23–25), and trauma from sexual assault (26).

Adapting existing interventions and developing new interventions can help address gaps in sexuality education for LGBTQIA+ youth. New digital interventions, including web- and text-based programs, have shown promise in improving identity self-acceptance, sexual health knowledge, communication skills, and contraceptive use during PV sex among LGBTQIA+ youth (27, 28). Studies describing these programs and their impacts provide valuable information to inform additional curricula that serve LGBTQIA+ youth.

Despite a growing number of programs designed to meet the sexual health needs of LGBTQIA+ youth, there is limited literature documenting how to tailor such evaluations. Standard evaluation practices may limit the ability to document impacts for LGBTQIA+ youth and even contribute to the marginalization of this population. To address this issue, we describe an LGBTQIA+-inclusive approach to evaluating *SafeSpace,* a mobile app-based sexuality education curriculum that was intentionally designed to resonate with LGBTQIA+ youth. We highlight specific strategies for centering the needs and experiences of LGBTQIA+ youth in all aspects of the program evaluation—from navigating Institutional Review Board (IRB) and funder requirements to recruitment and enrollment to assessing outcomes. These strategies can be broadly applied to tailor evaluations and strengthen the evidence base for programs that support the sexual health and well-being of LGBTQIA+ youth.

*SafeSpace* is a self-paced, mobile app-based 10-week program for youth 14 to 18 years old, assigned female or intersex at birth.^3^ The intervention was adapted from *Real Talk,* a mobile app publicly available in the Apple App Store that uses storytelling and technology to improve youth mental, emotional, and behavioral health (29). The *SafeSpace* program centers youths’ desire for stories from different youth perspectives and provides a sense of privacy (30). *SafeSpace* takes an LGBTQIA+-inclusive approach to all topics covered, including healthy relationships; identity and development; pregnancy and STI/HIV prevention; safety, communication and decision making; and accessing healthcare.

We are evaluating *SafeSpace* using a randomized control trial (RCT) design, which will allow us to measure attitudes, intentions, self-efficacy, and behaviors of participants who have access to *SafeSpace* compared to participants in the control condition, who have access to a general health app that does not include SRH information. Because 45 percent of youth who use *Real Talk* identify as LGBTQIA+, we are tailoring our recruitment efforts to ensure that at least half the sample identifies as LGBTQIA+ .^4^ We are also prioritizing youth of color and youth living in rural areas in the southeastern United States (31, 32).

## 2 Methods

### 2.1 Pilot implementation

From December 2022 to January 2023, we used paid social media ads to recruit 42 pilot study participants. Potential participants were invited to download *SafeSpace* from the Apple App Store and complete an eligibility screener within the app. Using an RCT design, eligible youth then completed a consent/assent form and baseline survey and were randomized to receive the intervention (*SafeSpace* SRH*)* or control (*SafeSpace* general health) app. Participants were randomized based on sexual orientation (LGBTQIA+, cisgender-straight), race and ethnicity (Hispanic regardless of race; non-Hispanic Black; and all other non-Hispanic non-Black races, such as White, Asian, Native Hawaiian or Pacific Islander, Indigenous American)^5^, and age (14–16, 17–18 years). We used Stata Version 16.1 to produce overall baseline prevalence estimates of demographic characteristics and sexual behaviors and test for differences in the distributions of sexual health behaviors by LGBTQIA+ status using chi-square tests. We focus on measures of sexual behavior particularly relevant to LGBTQIA+-inclusivity, but also include several other measures, like unprotected sex, to provide additional context.

### 2.2 Strategies to achieve LGBTQIA+-inclusivity

To ensure LGBTQIA+-inclusivity in the evaluation of *SafeSpace*, we used the following strategies: (1) staffing the project team with LGBTQIA+ researchers; (2) securing an IRB waiver of parental consent; (3) utilizing an LGBTQIA+focused recruitment approach and (4) developing and refining survey measures to reflect the experiences of LGBTQIA+ youth. To achieve the latter, we gathered feedback from equity experts, conducted cognitive interviews with LGBTQIA+ youth, and secured permission from the funder to omit required measures deemed non-inclusive. Together these strategies embed LGBTQIA+ equity at each stage of the evaluation, increasing the likelihood that LGBTQIA+ youth participants feel included, respected, and represented in this evaluation and enhancing our ability to document program impacts for this population.

#### 2.2.1 Staffing the project with LGBTQIA+ researchers

As a first step towards ensuring that LGBTQIA+ equity was central to the evaluation, we staffed the project with researchers who were members of the LGBTQIA+ community. By intentionally including LGBTQIA+ staff members, we ensured that our team had a critical eye towards LGBTQIA+ youth’s needs and perceptions and were able to identify what was needed to meet those needs throughout the evaluation. Additionally, these staff brought their knowledge as members of the LGBTQIA+ community, as well as credentials related to SRH, LGBTQIA+ psychology, gender and sexuality studies, and LGBTQIA+ health. While the impact of their insights on this project is immeasurable, these staff identified the necessity of each of the following steps taken to increase inclusivity.

#### 2.2.2 Securing IRB approval for consent procedures

Recognizing that requiring parental consent for study participation has unique implications for LGBTQIA+ youth, we requested and received a waiver of parental consent from the Child Trends IRB. We explained that requiring parental consent could result in unwanted disclosure about sexual and gender identity that could contribute to emotional distress for LGBTQIA+ youth. Further, sampling bias would increase if we only included youth with parental support, i.e., those with parental consent.

#### 2.2.3 Utilizing a LGBTQIA+ focused recruitment approach

To recruit LGBTQIA+ youth in our pilot study, we utilized two major social media platforms, TikTok and Instagram, to run paid advertisements (ads). Before launching the pilot, we conducted tests on four platforms—Facebook, Snapchat, TikTok, and Instagram. These tests aimed to identify the most effective targeting parameters, platforms, assets, keywords, and ad formats (such as Reels/Videos, Stories, and Feed) for reaching and engaging LGBTQIA+ youth and youth of color.

#### 2.2.4 Developing and refining LGBTQIA+-inclusive survey measures

To develop LGBTQIA+-inclusive survey measures, we incorporated feedback from the evaluation team, Child Trends LGBTQIA+ equity experts, and LGBTQIA+ youth. We used an iterative process to build consensus among these parties. The evaluation team led the initial measure development and refinement, specifically sexual orientation, gender identity, sexual behavior, and sexual agency measures. Relying heavily on insights from LGBTQIA+ staff, we first identified which questions needed revision or expansion. From there, we integrated findings of published research from The Trevor Project and other equity-focused institutions, including Child Trends’ resources on equity-centered survey design (33–38). We then proposed new measures and shared them with the LGBTQIA+ equity experts.

### 2.3 Equity experts

Two Child Trends LGBTQIA+ equity experts reviewed the new survey measures for inclusion, clarity, and respectfulness. The experts provided their feedback and suggested edits to the measures in written format or through video calls. The evaluation team applied the agreed upon edits to the survey and began cognitively testing the revised measures with LGBTQIA+ youth.

### 2.4 Cognitive interviews

We conducted cognitive interviews with seven LGBTQIA+ youth ages 14–18 who were recruited through social media advertisements testing (described above). Interviews were conducted via phone and lasted about 1 h. We presented interviewees with the newly developed questions, and they were encouraged to read the questions to themselves, describe their reactions, and discuss what they might consider when answering each question. Through this process, youth provided feedback and offered suggestions for clarity, comfort, and inclusiveness. In the few cases that feedback from youth contradicted feedback from the equity reviewers we prioritized feedback from youth after consulting with the evaluation team’s LGBTQIA+ staff.

### 2.5 Sex performance measure

This evaluation is federally funded by the Family and Youth Services Bureau’s Personal Responsibility Education Program—Innovative Strategies (PREIS), and as a PREIS grantee, we are required to collect specific demographic measures for performance measure reporting. We requested a waiver of the required measure for biological sex, which only has “Male” and “Female” response options. Child Trends IRB would not approve the survey instrument without including ‘Intersex’ as a response option, which was a compelling justification for our waiver.

## 3 Results

### 3.1 Consent procedures

The specific language used to obtain a parental consent waiver for minor participants is provided in Supplementary Material B. We described how requiring parental consent could result in unwanted disclosure of young people’s sexual or gender identity to parents, potentially resulting in emotional distress and undermine the scientific validity of the evaluation by contributing to selection bias. We also emphasized that (1) participating in *SafeSpace* involved no more than minimal risk to participants; (2) waiving parental consent did not adversely affect participants’ rights and welfare; (3) youth were required to assent/consent to each of the study requirements before they were enrolled in the study; and (4) youth were given the opportunity to skip intervention content and had access to resources pertaining to each of the curriculum topics after engaging with the material. Through this request, the evaluation team successfully obtained a parental consent waiver for participants ages 14–17. With this consent waiver, we helped to protect LGBTQIA+ participants by preventing potential identity disclosures.

### 3.2 Recruitment

Pre-pilot testing of ads informed the minimalistic design used for the pilot study recruitment campaign and helped us tailor ads to resonate with the differing experiences, identities, and interests of LGBTQIA+ youth by using LGBTQIA+ pride symbols, color palettes, and themes (see Supplementary Material A).

Pilot study ads performed well, particularly on Instagram. In total, we reached 469,878 youth^6^, resulting in 1,933 link clicks and 801,468 impressions. In total, 42 participants were enrolled in the pilot study, with more than half (61.9%) identifying as LGBTQIA+ .

### 3.3 LGBTQIA+-inclusive measures

The final measures fall within two domains: demographic characteristics (sexual orientation, gender identity, and sex assigned at birth) and sexual behaviors (sexual touching, rubbing genitals, oral sex, PV sex, anal sex, and sexting). In total, we included three demographic characteristic measures assessing sexual orientation, gender identity, and sex assigned at birth and 13 measures addressing lifetime and recent (past three months) sexual behaviors (Table 1). For each type of measure, we present themes from the internal equity reviews and cognitive interviews as well as baseline prevalence estimates from the pilot study (Table 2).

**Table 1:** Newly developed LGBTQIA+-inclusive measures.

Topic	Pilot study question(s)	
	Items	Response options
Demographic Characteristics		
Sex Assigned at Birth, Gender Identity, Sexual Orientation	What sex were you assigned at birth (which may or may not be different from your gender identity)?	Male
		Female
		Intersex
	Gender identity is how someone feels about their own gender. There are many ways a person can describe their gender identity and many labels a person can use.	Girl or woman
		Boy or man
		Nonbinary, genderfluid, or genderqueer
	Which of the following terms best describes your current gender identity?	
		I am not sure or questioning
		I don't know what this question means
		Decline to answer
		A gender not listed here [space for write-in]
	Sexual orientation is a person's emotional, romantic, and/or sexual attractions to another person. There are many ways a person can describe their sexual orientation.	Straight or heterosexual
		Gay or lesbian
		Bisexual or pansexual
	Which of the following best describes your sexual orientation?	Queer
		Asexual
		I am not sure or questioning
		I don't know what this question means
		Decline to answer
		An orientation not listed here [space for write-in]
Sexual Behaviors		
Types of Sexual Behaviors, past 3 months*	In the past three months, have you and another person touched each other's private parts for your or their pleasure? This includes fingering, hand jobs, or touching breasts.	Yes
		No
	Please only answer about sexual experiences you chose for yourself.	
	In the past three months, have you given or received oral sex? By oral sex, we mean a person's mouth touching another person's vagina, penis, or anus (butthole) for their own or their partners' pleasure.	
	Remember, only answer about times you willingly chose to participate.	
	In the past three months, have you had penis-vagina sex? By penis-vagina sex, we mean a penis goes inside a vagina.	
	Remember, only answer about times you willingly chose to participate.	
	In the past three months, have you rubbed your genitals or anus against another person's genitals or anus without penetration?	
	Remember, only answer about times you willingly chose to participate.	
	In the past three months, have you had sex using sex toys with another person? By sex using sex toys, we mean using vibrators, dildos, or butt plugs with another person for your or their pleasure?	
	Remember, only answer about times you willingly chose to participate.	
Sexting, ever	Have you ever sent a sexual photo or video of yourself to another person? Sending or receiving sexual photos or videos is also called “sending nudes” or “sexting.”	Yes
		No
	Remember, only answer about times you willingly chose to participate.	
	Has another person ever sent you a sexual photo or video of themselves?	
	Has someone ever asked you to send them a sexual photo or video of yourself?	

* Only measures for the “past three months” are displayed here. However, at baseline, participants were asked questions using this language to measure their sexual behavior “ever” and in the “past 3 months.” These measures can be edited to measure sexual behavior at differing timepoints as needed.

**Table 2:** Feedback for LGBTQIA+-inclusive measures.

Topic	Original question(s)	Feedback from internal equity reviews	Feedback from cognitive interviews
Demographic Characteristics			
Biological Sex, Gender Identity, Sexual Orientation	What is your sex?	Consider Child Trends approved sex assigned at birth question that includes intersex as a response option and clarifies sex as distinct from gender identity.	NA
	MARK ONLY ONE ANSWER		
	• Male • Female		
	Which of the following best describes your gender identity (which may be different than your sex assigned at birth)?	Add response options including genderqueer, unsure, and a write-in response for “a gender not listed here”.	NA
	• Female (Girl/Woman) • Male (Boy/Man) • Non-binary, Gender fluid, or Gender expansive • A gender not listed here • Prefer not to say	Remove sex-related terms and stick to gender identity terms.	
	Which of the following best describes your sexual orientation? • Straight (not gay) • Gay or lesbian • Bisexual or pansexual • Asexual • I am not sure yet • An orientation not listed here	Add response options including “I don't know what this question means” and a write-in option for “an orientation not listed here”.	Participants liked an addition of a “queer” response option.
		Add a definition of sexual orientation to the question.	Participants liked definitions of sexual orientation that included the phrase “people use many labels.”
		Remove parenthetical descriptor for “straight”.	
Sexual Behavior			
Sexual Behavior, past 3 months	The next questions are about vaginal sex. By vaginal sex, we mean a penis in a vagina.	The term “penis-vagina sex” might be more familiar and accessible to adolescents and will be more specific to the exact type of sex than “vaginal sex.”	Continue to include a qualifier, “Please only answer about sexual experiences you chose for yourself,” about not answering activity questions about non-consensual experiences.
	In the past 3 months, have you had vaginal intercourse?		
	The next questions are about anal sex. By anal sex, we mean a penis (not a sex toy) goes inside an anus (butthole).	Use anatomical words for genitalia instead of private parts, to be more specific and sex positive.	Consider how to include asexual people when defining sex as “for pleasure.”
	In the past 3 months, have you had anal sex?		
	The next questions are about oral sex. By oral sex, we mean a mouth touches a vulva, penis, or anus (butthole).	Use anatomical words consistently, for example, vagina instead of vulva.	Both colloquial and anatomical words for genitalia were humorous, but understandable. Vagina was preferred over vulva. “Areas of pleasure” and “private parts” may be acceptable too.
	In the past 3 months, have you had oral sex?		
	Have you ever touched someone's private parts with your hands?	Remove the parenthetical comment descriptor “not a sex toy” in the definition of anal sex.	Sexual touching is an important type of sex to ask about.
	Have you ever let someone touch your private parts with their hands?		The oral sex definition should include giving and receiving sex.
	In the past 3 months, have you had vulva-to-vulva sex? By vulva-to-vulva sex, we mean when a vulva touches another vulva.		Inclusion of the term “without penetration” in the definition of sexual rubbing is confusing.
	The next question is about having sex using sex toys like vibrators, dildos, or butt plugs with another person. In the past 3 months, have you had sex using sex toys with another person?		
Sexting, ever	The next questions are about sending or receiving sexually explicit text, photo, or video messages (also called “sexting”).	Different people may define “sexually explicit” in different ways.	NA
	Remember, only answer about times you willingly chose to participate. In the past 3 months, have you: Sent a sexually explicit photo or video message to another person? • Yes • No		
	Received a sexually explicit photo or video		
	message from another person? • Yes • No		

#### 3.3.1 Demographic characteristic measures

##### 3.3.1.1 Themes from internal equity reviews

Our equity reviewers provided feedback that incorporated best practices from the literature and examples from well-respected organizations in the field (Table 2). Themes included (1) shifting away from binary measures of sex, gender identity, and sexual orientation and (2) enhancing youth comprehension. Regarding gender, an equity reviewer stated, “there is some evidence that trans folks will choose their sex here accidentally, and I think your sample is old enough to understand girl/woman and boy/man, so I would drop the female and male.” To improve our sexual orientation question, reviewers advised we use question wording from the Trevor Project’s definition of sexual orientation (38).

They also suggested that the parenthetical “not gay” addition to the “straight” response option “may be a holdover from previous times and less necessary now” and that “it also feels like it forces a binary.” Lastly, the reviewers recommended adding write-in options to allow participants to describe their identities in their own words as well as a response option of “I don’t know what this question means.”.

##### 3.3.1.2 Themes from cognitive interviews

Young people participating in the cognitive interviews had positive feedback to share about the demographic measures (Table 2). They indicated that the measures were easy to understand, comprehensive, and inclusive. Although one participant suggested the term “sexual identity” might be more accurate than the term “sexual orientation,” all other participants preferred “sexual orientation” so we retained that language.

##### 3.3.1.3 Pilot study baseline results

Participant demographics are shown in Table 3. Similar proportions of participants identified as bisexual, pansexual, or queer (40.5%) and straight (38.1%). About one in ten (11.9%) indicated that they were questioning their sexual identity, and 4.8 percent identified as lesbian or gay, and asexual each. Most participants identified as a girl or woman (85.7%), followed by non-binary, gender nonconforming, or something else (11.9%). One (2.4%) reported questioning their gender identity.

**Table 3:** Pilot study demographics (*N* = 42).

	*N*	%
LGBTQIA+	26	61.9%
Cis-Straight	16	38.1%
Sexual Orientation		
Straight	16	38.1%
Lesbian or gay	2	4.8%
Bisexual, pansexual, or queer	17	40.5%
Asexual	2	4.8%
Questioning	5	11.9%
Don't know or prefer not to say	0	0%
Gender Identity		
Girl or woman	36	85.7%
Boy or man	0	0%
Non-binary, gender nonconforming, or something else	5	11.9%
Questioning	1	2.4%
Don't know or prefer not to say	0	0%
Race/Ethnicity		
Non-Hispanic Black	16	38.1%
Non-Hispanic Non-Black	16	38.1%
Hispanic	10	23.8%
Age		
14–16	18	42.9%
17–18	24	57.1%

#### 3.3.2 Sexual behavior measures

##### 3.3.2.1 Themes from internal equity reviews

For the sexual behavior measures, equity reviews provided feedback related to enhancing (1) youth comprehension, (2) consistency, and (3) gender inclusiveness (Table 2). For example, regarding PV sex, equity reviewers noted, “from a say-what-you-mean perspective, penile-vaginal sex is probably what you are talking about. I have seen the term penis/vagina sex used, which might be easier for youth since the adjectives penile and vaginal may be less familiar than the nouns.” Similarly, in response to a measure about vulva-to-vulva sex, the equity reviewers were “not sure everyone knows the term vulva and apart from this you seem to be using vagina instead, which is probably more commonly understood.” Furthermore, to be more inclusive, they suggested referring this activity as “genital-to-genital sex” when defining non-penetrative types of sex. Additionally, the reviewers noted that specifying that anal sex excludes sex toy use could underestimate the prevalence of this activity given that butt plugs are sometimes used to prepare for anal sex. Reviewers suggested instead that we remove the parenthetical phrase “not a sex toy” from the definition of penetration in anal sex.

##### 3.3.2.2 Themes from cognitive interviews

Themes from the cognitive interviews addressed (1) the comprehensibility and accuracy of the language, and (2) the inclusivity of our language and definitions (Table 2). Some comments helped us to add clarity to our definitions of in-person sexual activity. For example, one participant noted that the phrase “experienced oral sex” could be interpreted as only receiving oral sex and that we should revise it to explicitly measure both receiving and giving oral sex. Other comments affirmed that the measures would be well-understood by young people. For example, regarding our use of both medical and colloquial terminology for genitalia, a participant noted “for some 14-year-olds, [medical terminology] might be confusing to some, particularly anus” but “you put butthole in parentheses, so you’re giving them another word of what it is.”

Young people’s feedback also enhanced the inclusivity of our measures. In particular, one participant highlighted that “for AFAB [assigned female at birth] people who may identify [not as a girl or woman], the word vagina might make them uncomfortable or be triggering. Vulva might be a softer way to phrase it. Vagina [is] also used more as slang [for entire genital area].” This individual suggested using a phrase such as “areas of sexual pleasure,” yet we proposed “private parts” as an alternative to other participants who responded positively to this phrase. Another participant also suggested we alter our language to include asexual youth, noting we should change “wording to [account for] sexual experiences you are willing to do or are comfortable with but not necessarily for pleasure.” Finally, interviewees appreciated the language reminding respondents to report only on wanted sexual experiences, noting that this reminder was “very affirming.”

##### 3.3.2.3 Pilot study baseline results

Overall, 42.9 percent of all participants had engaged in any type of in-person sexual activity in the past three months; participants primarily engaged in sexual touching (40.5%) and oral sex (35.7%); only 4.8 percent engaged in anal sex. Forty-six percent of LGBTQIA+ participants had engaged in any type of in-person sexual activity in the past three months, compared to 37.5 percent of cis-straight participants. Seven percent of LGBTQIA+ participants had anal sex in the past three months whereas no cis-straight participants reported this behavior. Similarly, about one-fifth (19.2%) of LGBTQIA+ participants reported having sex using sex toys whereas no cis-straight participants reported this behavior. None of these differences by LGBTQIA+ status were statistically significant.

Most pilot participants had ever been asked to send a sext (90.4%) and had received a sext (85.4%); more than half had sent a sext themselves (61%) (Table 4). A higher proportion of LGBTQIA+ participants had been asked to sext, received a sext, and sent a sext compared to cis-straight participants yet the differences were not statistically significant.

**Table 4:** Baseline sexual behaviors by LGBTQIA+ Status (*N* = 42).

	Full sample		LGBTQIA+		Cis-straight		*p*-value
	*N*	%	*N*	%	*N*	%	
In-person sexual behavior, past 3 months							
Any type of in-person sexual activity in the past 3 months	18	42.9%	12	46.2%	6	37.5%	0.582
Sexual Touching	17	40.5%	11	42.3%	6	37.5%	0.758
Oral Sex	15	35.7%	10	38.5%	5	31.3%	0.636
Penile-Vaginal Sex	11	26.2%	7	26.9%	4	25.0%	0.891
Sexual Rubbing	8	19.5%	6	24.0%	2	12.5%	0.365
Anal Sex	2	4.8%	2	7.7%	0	0%	0.256
Sex with Sex Toys	5	11.9%	5	19.2%	0	0%	0.062
Sexting behavior, ever							
Ever been asked to send someone a photo or video	37	90.4%	25	96.2%	12	80.0%	0.446
Any sexting—recipient	35	85.4%	24	92.3%	11	73.3%	0.098
Any sexting—sender	25	61.0%	17	65.4%	8	53.3%	0.093
Condom and contraceptive use, past 3 months							
Penile-vaginal or anal sex without using a condom every time	10	23.8%	6	23.1%	4	25.0%	0.887
Penile-vaginal sex without using pill, shot, patch, ring, IUD, implant, or condom every time	9	21.4%	6	23.1%	3	18.8%	0.740

We also collected reports of PV sex without contraception and condomless PV or anal sex in the last three months. Less than a quarter of all participants had PV sex without contraception (21.4%) or had engaged in condomless PV or anal sex (23.8%) in the past three months. The differences between cis-straight and LGBTQIA+ participants’ engagement in PV sex without contraception in the past three months was not statistically significant, nor were the differences between participants in rates of condomless PV or anal sex by sexual orientation and gender identity.

## 4 Discussion

The recent development of innovative LGBTQIA+-inclusive sex education programs helps to fill sexuality education gaps for LGBTQIA+ youth. However, evaluations of these programs must accurately capture the experiences of these populations to ensure LGBTQIA+-inclusive programming is evidence-based and can be widely scaled up. This study shows how we incorporated an integrated approach to develop and pilot test a rigorous LGBTQIA+-inclusive evaluation of the *SafeSpace* adolescent sexual health program.

Our pilot study demonstrates the feasibility and effectiveness of using social media ads with tailored graphic designs to recruit a sample of youth participants with diverse sexual orientations, racial identities, and gender identities into a mobile app-based intervention. Notably, almost two-thirds of pilot sample participants were LGBTQIA+ and three-quarters were Black and/or Latinx. Because the types of platforms youth use are constantly changing (39, 40), social media recruitment must be tailored to meet the needs of youth. For example, previous studies recruited large samples through Facebook (41–44) and Instagram (33), while this study also used TikTok, which was launched in 2016 (45).

This study also highlights the feasibility of obtaining a parental consent waiver—an important study enrollment practice for evaluation of sex education programs. Parental consent requirements for minors to participate in research can disproportionately exclude LGBTQIA+ youth who have not disclosed their identity to their parents or who are living with chosen family (46). Given that parental consent requirements could lead to selection bias in which LGBTQIA+ youth with parental support are overly represented, we intentionally sought and received exemption from a parental consent requirement by clearly documenting the potential harms to participants and threats to validity.

By incorporating insights from LGBTQIA+ youth, LGBTQIA+ staff, and equity experts, we created survey measures that expand beyond a heteronormative, cisnormative, and risk-oriented prioritization of PV sex (38, 47, 48). These measures better reflect, respect, and affirm the experiences of LGBTQIA+ program participants who may otherwise feel alienated by questions not inclusive of their sexual experiences (49). However, our pilot study baseline findings indicate that these measures better capture the variety of sexual experiences of all youth. Both LGBTQIA+ and non-LGBTQIA+ youth reported engaging in non-PV types of in-person sexual activity, including sexting, sexual touching, genital rubbing, anal sex, and oral sex. As such, we normalize and validate all adolescents’ sexual desires and behaviors. Moving away from measuring only PV sex shifts the adolescent sexual health field from a sex-negative framework focusing on sex that carries a reproductive risk and towards a sex-positive framework that affirms sex for the purpose of pleasure, connection, and wellbeing. which can be developmentally appropriate for adolescents (50). This shift toward sex-positivity allows researchers to document aspects of both LGBTQIA+ adolescents’ and cis-straight adolescents’ sexual wellbeing, not only their sexual health (51).

By including a broader set of measures of sexual behavior, we captured a substantially higher rate of sexually activity from 26 percent who engaged in recent PV sex, to 42 percent who engaged in any type of in-person sexual activity in the past three months. Estimating higher rates of in-person sexual activity also has implications for evaluations’ ability to measure the impact of programming on STI prevention, consent, and other sexual health outcomes. The high rates of PV sex without contraception and condomless PV or anal sex at baseline—21 percent and 24 percent, respectively—reflect substantial exposure to unintended pregnancy and STIs for both LGBTQIA+ and cis-straight participants. Strikingly, almost all youth (both LGBTQIA+ and cis-straight) who reported PV or anal sex at baseline indicated at least one incident of recent sex without contraception or condoms, which points to the need for comprehensive and inclusive sexual health programming.

One notable finding from the pilot was the high percentage of youth engaging in sexting, with 61 percent reporting they ever sent a sexual photo of themselves to another person and 85 percent reporting receipt of sexual photos. These pilot results are much higher than a recent meta-analysis finding that almost 20 percent of youth report sending sexts and more than one-third report receiving sexts (52). The difference in rates may be due to the timeframe (we measured ever vs. recent sexting and measured “sexual photos” instead of “nude photos”).

There are several limitations to this study. First, this is a pilot study so the sample size is small; as such significance testing may be unreliable. Additionally, while we took care to describe how *SafeSpace* participants would take the evaluation surveys, our cognitive interviewees were presented with the survey items in a word document on a call with study team members, while the participants filled the survey out online independently. This different format may have impacted how they interpreted the questions and responses. Finally, we oversampled LGBTQIA+ youth to understand their unique perspectives and experiences. As such the results are not generalizable. Instead, we hope to demonstrate the feasibility of conducting an LGBTQIA+-inclusive evaluation.

## 5 Conclusion

Our findings indicate that leveraging the lived experiences of LGBTQIA+ staff and incorporating input from equity reviewers and LGBTQIA+ youth is a feasible and effective approach to designing and implementing LGBTQIA+-inclusive evaluations of sexual health programming. Our study highlights the types of strategies that can successfully be used to conduct LGBTQIA+-inclusive evaluations to better support the sexual health of both LGBTQIA+ and cis-straight youth. Future sexual health program evaluations should incorporate such strategies to inclusively, respectfully, and more accurately capture the experiences and identities of LGBTQIA+ youth. In doing so, program evaluations can document the impact of and need for sexuality education that is inclusive, affirming, and that promotes sexual wellbeing for all.

## Data Availability

The datasets presented in this article are not readily available because we did not receive IRB approval to make the data publicly available. Requests to access the datasets should be directed to Elizabeth Cook, ecook@childtrends.org.

## Footnotes

^1^We use the definition of sexuality education that Breuner et al use, which includes content on sexual health topics such as sexually transmitted infections, contraception, sexual activity, and reproductive rights; sexual and romantic relationships; anatomy and reproduction; and personal identity topics such as sexual orientation and gender identity (1).

^2^LGBTQIA+ is an acronym for lesbian, gay, bisexual, transgender, queer/questioning, intersex, asexual, and other marginalized sexualities and genders. We use the full acronym to reflect the diversity in this population. However, when referring to other programs we follow the acronym used by that organization.

^3^We chose to include AFAB and intersex participants for several reasons. First, we are interested in understanding contraceptive use as a key outcome, as well as clinic access, which tend to be more relevant to AFAB youth. Additionally, we wanted reliable data on contraceptive use, and male reporting of partner’s contraceptive use is less accurate than method users themselves. Further, as *SafeSpace* was developed from *Real Talk*, we aimed for our study population to reflect its users. Over 60% of *Real Talk* users identify as girl/woman or non-binary/gender non-conforming. Additionally, while there is large variability within the intersex population, we included intersex people in the evaluation to (1) include as many people as possible who may use contraception and be able to get pregnant in our sample without being overly intrusive with questions about their specific condition, and (2) we wanted to include as many LGBTQIA+ people as possible. As such, intersex people are eligible to participate even though some of them are not able to become pregnant.

^4^MyHealthEd, Inc. Real Talk Mobile App Analytics Data. 2019. [Unpublished data].

^5^This *SafeSpace* program evaluation has an intentional focus on Hispanic and non-Hispanic Black youth, so all other youth are grouped together. We used these categories in our recruitment approach, requiring at least 50 percent of our sample to be Hispanic or non-Hispanic Black youth. We accepted all non-Hispanic, non-Black youth who met the inclusion criteria to make up the rest of the sample.

^6^Reach is a measure used in the social media marketing industry to represent the number of accounts (people) that saw an advertisement at least once.
