# Supplementary material for: Strategies to develop an LGBTQIA+-inclusive adolescent sexual health program evaluation
Source: Front Reprod Health. 2024 Mar 22;6:1327980. doi: 10.3389/frph.2024.1327980 (PMC11000634; doi:10.3389/frph.2024.1327980)
Supplement: Supplementary file 1 [file Datasheet1.pdf]

Supplementary Materials

1 Waiver of Parental Consent for Youth Ages 14-17

1.1 Waiver of Parental Consent Request

A waiver of parental consent is being requested for participants in this evaluation based on the following reasons:

1. We believe that participating in this intervention involves no more than minimal risk to the subjects. As stated earlier in the application, we recognize that the topics of our questions may make participants uncomfortable. Since we are particularly interested in recruiting LGBTQ+ youth to ensure the study is inclusive of all gender identities and sexual orientations, we believe that requiring parental consent will cause youth to decline or opt out of participating in sexual health research to avoid disclosing their gender identity, sexuality, interest in sexual health education, and/or previous sexual activity to their parents (1–3). In order to protect the privacy of our participants and prevent mistimed disclosures, we believe that the parental consent waiver is critical for completing the pilot study.

2. We believe that the rights and the welfare of participants will not be adversely affected by waiving parental consent. Participants ages 14-17 will be required to give assent but will otherwise receive the same information as participants 18 and over. One potential concern is that youth will be unable to make an informed decision to participate in the study and will feel pressured to participate. Research has found that youth between the ages of 14 and 17 understand their rights as participants and the principles of informed consent when self-consenting to sexual health research (3). To ensure that youth understand their rights, the consent form, and the purpose of the study, all youth will be required to complete a set of review questions before they are allowed to give consent or assent to participate. If the participant does not appear to comprehend the assent/consent procedures or the research, they will not be included in the pilot study.

3. We believe that the pilot study should only be carried out with this parental consent waiver. Overall, LGBTQ+ youth are underrepresented in sexual health research (4,5). By only conducting the pilot study with young people whose parents would provide consent, we would be excluding an important portion of our sample, which would result in biased, and therefore, invalid data that would not be informative to the overall purpose of our project or to the field (5). Further, without the parental consent waiver, the pilot study could ultimately incite familial strife; untimely discussions about gender, sexuality and identity; and emotional distress. For these reasons, a waiver of parental consent is imperative for this pilot study.

4. Although we do discuss sensitive topic in the survey and Safe Space App Curriculum, youth will be given the opportunity to access resources pertaining to each of the curriculum topics after engaging with the material. If the participant is interested in learning more about a topic or would like to find support near them, they will be given a

link to resources on topics such as bullying, relationship violence and reproductive health. Further, the curriculum developer has created a trigger warning feature that indicates to participants that a module contains potentially distressing information, provides a direct link to the homepage and allows the participant to skip the module.

1.2 References for Waiver of Parental Consent Request

1. Smith AU, Schwartz SJ. Waivers of parental consent for sexual minority youth. Accountability in Research. 2019 Aug 18;26(6):379–90.

2. Macapagal K, Coventry R, Arbeit MR, Fisher CB, Mustanski B. “I Won’t Out Myself Just to Do a Survey”: Sexual and Gender Minority Adolescents’ Perspectives on the Risks and Benefits of Sex Research. Arch Sex Behav. 2017 Jul 1;46(5):1393–409.

3. Fisher CB, Arbeit MR, Dumont MS, Macapagal K, Mustanski B. Self-Consent for HIV Prevention Research Involving Sexual and Gender Minority Youth: Reducing Barriers Through Evidence-Based Ethics. Journal of Empirical Research on Human Research Ethics. 2016 Feb 1;11(1):3–14.

4. Fisher CB, Mustanski B. Reducing Health Disparities and Enhancing the Responsible Conduct of Research Involving LGBT Youth. Hastings Center Report. 2014;44(s4):S28–31.

5. Mustanski B. Ethical and Regulatory Issues with Conducting Sexuality Research with LGBT Adolescents: A Call to Action for a Scientifically Informed Approach. Arch Sex Behav. 2011 Aug 1;40(4):673–86.

2 Recruitment Images for Instagram

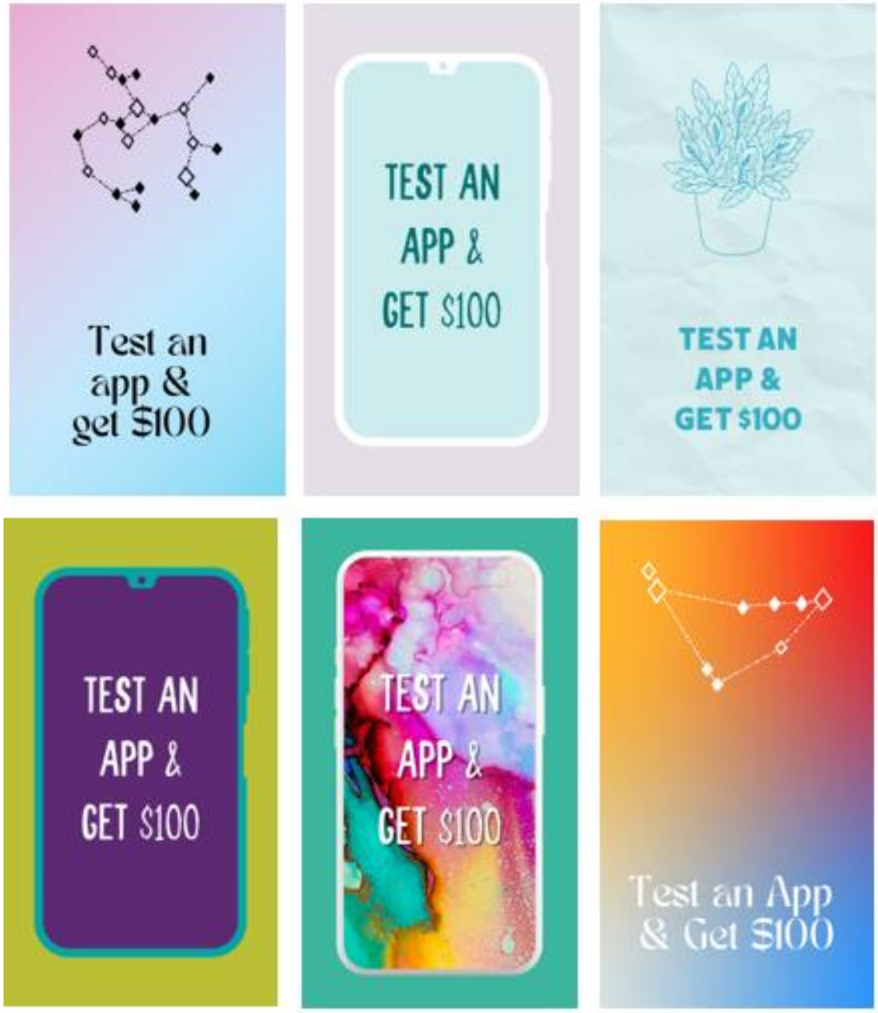

Supplementary Figure 1. Each of these images were posted individually on Instagram as advertisements to recruit participants for the SafeSpace program.

3 Ad Captions for Social Media Recruitment

3.1 Ad Captions for Recruitment on Instagram

The following captions were used on advertisements to recruit participants for the SafeSpace program on Instagram.

- We need your help! 📢 We're creating a new app made just for teens, and we want to hear your real, unfiltered thoughts. Download the app today! 📱🏳️
#LGBTQ #ReproductiveHealth #SexEdForAll #SexEducation #Health

- You heard it: get up to $100 for helping us test an app made just for young people like you. 🙌 Download the app today! 📱🏳️‍🌈
#LGBTQ #ReproductiveHealth #SexEdForAll #SexEducation #Health

- We're looking for young women to help test our new app! ✨📱 Join us today if you're:
- 14 to 18 years old
- Black or Latinx
- LGBTQ+
- Or living in a rural area
#LGBTQ #ReproductiveHealth #SexEdForAll #SexEducation #Health

- Spots are filling fast! There's still time to help us test a new app – made just for teens – and get up to $100 just for sharing your real, unfiltered thoughts. Sign up now and download the app! ✨🏳️‍🌈
#LGBTQ #ReproductiveHealth #SexEdForAll #SexEducation #Health

3.2 Ad Captions for TikTok

The following captions were used on advertisements to recruit participants for the SafeSpace program on TikTok. TikTok ads cannot include emojis or hashtags and can only be up to 100 characters, including spaces, so these captions are considerably shorter than the other social media platforms for this reason.

- We need your help—we're creating a new app made just for teens, and we want to hear your thoughts!

- You heard it: get up to $100 for helping us test an app made just for young people like you!
